# The effect of priming on fraud: Evidence from a natural field experiment

**DOI:** 10.1111/ecin.13088

**Published:** 2022-04-30

**Authors:** Parampreet Christopher Bindra, Graeme Pearce

**Affiliations:** ^1^ Department of Public Economic Theory University of Innsbruck Innsbruck Austria; ^2^ Department of Economics University of Exeter Business School Exeter UK

**Keywords:** credence goods, field experiments, fraud, priming

## Abstract

We present a natural field experiment to examine if priming can influence behavior in a market for credence goods. 40 testers took 600 taxi journeys in Vienna, Austria, and using a between–subject design we vary the script they spoke, each designed to prime either honesty, dishonesty, or a competitor. We find that the honesty prime increases taxi fares by 5.5% relative to a baseline, the result of overcharging rather than overtreatment. Priming dishonesty and a competitor have no impact on fares. We find that the effects of priming on behavior are likely to be small compared to information asymmetries.

## 1 INTRODUCTION

Markets for credence goods are characterized by informational asymmetries between consumers and sellers. For example, a doctor is more informed than the patient about the quality of care the patient needs in order to maximize their health outcomes, a car mechanic is employed by an uninformed motorist in order to determine if their car needs a new engine, and taxi drivers know the quickest and most cost efficient routes for their passengers (Darby & Karni, 1973). The problem arising in each of these examples is that the informational asymmetry puts the seller at a strategic advantage because the consumer is unable to observe the quality of the good they have received ex post. This creates a strong material incentive for the seller to behave dishonestly, but profitably, at the expense of the consumer (Dulleck & Kerschbamer, 2006).

The most well studied inefficiencies that arise in markets for credence goods are *overtreatment* and *overcharging* (Darby & Karni, 1973; Dulleck & Kerschbamer, 2006). The first, *overtreatment*, involves the seller providing a higher quality of work than is necessary in order to maximize their own profits. For example, car mechanics can replace an engine when only a tune up is required, or taxi drivers can take passengers on extended detours in order to increase a fare. The second, *overcharging*, involves sellers charging customers for a higher quality than is actually provided, or for a service that has not been supplied. For example, hospitals can charge patients for medicines they have not received, or taxi drivers can add unjustifiable surcharges to the meter when they are not necessary. As Dulleck and Kerschbamer (2006) highlight, although overtreatment reduces consumer welfare to the benefit of the seller, overcharging represents a direct transfer of utility from consumers to sellers.

Different market structures encourage different types of inefficiency, and the majority of previous empirical work has focused on testing the theoretical predictions of Dulleck and Kerschbamer (2006). For example, Balafoutas et al. (2013) use a field experiment to explicitly test the role of informational asymmetries, and find that the extent of overtreatment escalates as the informational disparity increases. Schneider (2012) conducted a field experiment to examine the extent of overtreatment in the market for car repairs, as well as the role played by reputation. Interestingly, Schneider finds that both overtreatment and undertreatment are widespread, but finds limited evidence that reputational concerns are important, possibly because of customers inability to assess service quality. Others have examined potential behavioral explanations for the deviations from the theoretical predictions, such as the importance of heterogeneity in social preferences (Kerschbamer et al., 2017), and the phenomenon of ‘second degree’ moral hazard (Balafoutas et al., 2017).^1^ The study of behavioral explanations and solutions to market inefficiencies has grown prominent, as in many markets it may be impossible to achieve the structure required to rule out various types of fraud, or such institutional changes may be costly or difficult to implement.^2^

The purpose of this paper is to gain further insight into how fraudulent behavior can be reduced. We focus on priming, a common technique used in psychology and used first by Karl Lashley in 1951 and was used to “indicate the influence of a previous stimulus elaboration on the evaluation of something”. When studying priming, a researcher introduces a stimuli (a “prime”) in order to activate social knowledge structures that can impact people's behavior outside of their awareness and control (Bargh, 2006). Once primed, individuals are able to recall certain memories more easily than others, which in turn leads to related or connected information to be activated at the same time. As economic agents' decisions are determined by the interplay of their preferences and beliefs, with preferences typically assumed to be given, one interpretation is that priming influences behavior through an individuals' beliefs. However, priming differs from ‘standard’ strategic effects in the sense that it is intended to impact belief formation by influencing the manner in which information in subsequent decisions is processed (Posten et al., 2014). Hertel and Fiedler (1998) define priming as the “procedural feature that some previously activated information impacts on the processing of subsequent information” (Hertel & Fiedler, 1998, p. 50). Previous recent work on priming in economics has been conducted in the laboratory and has examined the effect of primes designed to promote cooperation (Chen et al., 2014; Drouvelis et al., 2015) and competitiveness in women (Balafoutas et al., 2018). These studies used a range of techniques. For example, Drouvelis et al. (2015) primed subjects to cooperate by asking them to complete word searches where the hidden words were all pro–social, before taking part in a public goods game. Chen et al. (2014) used pictures and photos of the subject's University to prime a positive sense of group identity and encourage in–group favoritism. Balafoutas et al. (2018) found that they could reduce the gender gap in competitiveness if they first asked subjects to write an essay about feeling powerful. Cohn et al. (2015) used animated charts of a fictitious stock market as a prime to examine how ‘boom’ and ‘bust’ scenarios can impact the decisions of financial professionals. Cohn et al. (2017) implemented a questionnaire prime to understand how professional identities impact the behavior of bankers. Balafoutas et al. (2020) use self–reflection techniques to explore the social behavior of prison inmates. Similar findings have been reported in the study of risk preferences (Erb et al., 2002), trust (Burnham et al., 2000), reciprocity and altruism (Cappelen et al., 2011; McKay et al., 2010).^3^ Shu et al. (2012) examine the impact of priming on honest behaviors in both a laboratory and field setting, and find that by asking subjects to sign before completing a form, in comparison to after, dishonest behavior is reduced. However, Kristal et al. (2020) failed to replicate this result.

Priming has previously been found to successfully change how people think and behave (Balafoutas et al., 2018), and our motivation for examining priming is that it has the potential to be a near costless intervention that can result in behavioral change. Our motivation is therefore similar to that of the norm–nudge literature where simple, low cost interventions have been applied in a variety of settings to help people make ‘better’ decisions. The literature on nudges has shown how people can be encouraged to save more for retirement (Bernheim et al., 2015), stop smoking, and even take their medications (see Bicchieri and Dimant (2019) and Münscher et al. (2016) for two recent comprehensive reviews). Nudges have also been used to remind people what behaviors are appropriate in a given situation, such as appropriate driving behaviors (Guardian, 2013), or when not to prescribe antibiotics (Hallsworth et al., 2016). In many cases ‘norm–nudges’, the provision of social information to remind people how they should behave, have been used to promote pro‐social behaviors. However, as discussed in Bicchieri and Dimant (2019) and recently found by Chen et al. (2021), norm–nudges are not always successful and can even ‘backfire’. As Bicchieri and Dimant (2019) outline in a comprehensive discussion paper, norm–nudging will be ineffective when behavior is determined by preferences that are unconditional on the behavior of others, or when behavior is motivated by a moral rule.^4^

We conduct a natural field experiment (Harrison & List, 2004) to examine the fraudulent behavior of taxi drivers in Vienna, Austria. We employed 40 testers to take a total of 600 individual taxi journeys. Following the methodology of Balafoutas et al. (2013), testers took journeys in groups of four, with each tester in a group catching a taxi from the same location and going to the same destination within (approximately) a 1 minute interval of each other. They each carried a GPS trip logger, and documented detailed information about the journey and charges associated with the taxi fare. This enables us to observe precisely how the driver defrauds the passenger: comparing GPS tracker data allows us to identify unnecessary detours, and therefore the level of overtreatment between treatments, whereas additional charges added to the taxi meter let us examine the extent and intensity of overcharging. For every journey, the tester first signaled to the driver that they were foreign and that they did not know the way to their destination, ensuring that the ride constitutes a credence good (Balafoutas et al., 2013; Darby & Karni, 1973). Testers then spoke a simple script targeted to prime the driver with honesty, dishonesty, or market competition. In doing so, we examine the effect of priming on fraudulent behavior when the informational advantage of the seller over the consumer is largest, and in a context where we would expect fraud to already be present. Thus, we have the opportunity to implement treatments that could both reduce or increase fraud.

Using a 4 × 1 between–subject design, the experiment exogenously varies the script spoken by the testers. In a *Baseline* treatment, the testers only signaled they were a foreigner and did not prime the driver. This serves as a control to which we can compare the other treatments. Using an *Honesty* treatment, testers inform the driver that they have heard about a study (namely Balafoutas et al. (2013)) in which 80% of taxi drivers were found to behave honestly, saying to the driver, “Did you hear about that study where researchers found that around 80% of taxi drivers were shown to behave honestly toward passengers, always taking them on the cheapest route? I read about it on the internet.” In a *Dishonesty* treatment, testers provide drivers with the exact same information as the *Honesty* treatment, but instead emphasize that 20% of drivers were found to behave dishonestly, “Did you hear about that study where researchers found that around 20% of taxi drivers were shown to behave dis‐honestly toward passengers, taking them on more expensive routes than necessary? I read about it on the internet.” These treatments are similar in spirit to the literature examining valence framing effects (Levin et al., 1998), where information cast in a different light can have vastly different consequences for behavior, and how the re‐description of a problem cast in positive or negative light influences information processing (Liberman et al., 2004; Ward et al., 1997). Finally, motivated by the prediction of Dulleck and Kerschbamer (2006) that competitive conditions will increase overcharging, we implement an *Uber* treatment, where testers mentioned that a competitor's price for the same journey (an Über taxi) seems cheap. In this treatment testers said, “I checked the Uber price online and it seemed cheap.”

We report a number of observations. First, we find that taxi fares are around 5.5% larger when the driver receives the *Honesty* prime in comparison to a baseline fare. Our analysis reveals that the fare increases in the *Honesty* treatment are a consequence of drivers increasing the amount of fraudulent overcharging, rather than being due to increases in overtreatment. Despite the *Dishonesty* treatment conveying the exact same information to drivers as the *Honesty* treatment, it is found to have no discernible effect on the drivers' behavior in comparison to a baseline. We explain these findings as potentially being a consequence of the primes altering the drivers' belief about the probability they will be caught defrauding the passenger. There is no noteworthy effect of our *Uber* prime although the fares are significantly higher at the 10% level compared to a baseline.

Second, we find no significant treatment effects on the amount of overtreatment, with the primes having no effect on journey lengths. Importantly, this does not imply that the drivers are not overtreating our testers, but instead that overtreatment is constant between treatments. As the informational asymmetry between the passengers and drivers is high, we expect overtreatment to be prevalent, as in Balafoutas et al. (2013). This is similar to the findings of Balafoutas et al. (2017), who report evidence of increased overcharging by drivers in Athens, but no effect on overtreatment, when a passenger informs the driver they are claiming the fare from their employer. In order to examine this, we determine how the effects of our primes compare to the effect of a knowledge asymmetry between driver and passenger. We do this by examining how our testers are treated relative to a hypothetical perfectly informed passenger ‐ one who, given they themselves are an expert, would be taken on the shortest ‘optimal’ route and charged the associated fare. We find that the testers in our experiment faced no overtreatment on average, despite fares being approximately 44% higher than the fare associated with the optimal route. The drivers took our testers on journeys that are indistinguishable from the optimal route, however in all treatments our testers face a significant level of overcharging. As might be expected, we find that the effect of our primes is small in comparison to the effect of knowledge asymmetry between buyers and sellers.

This study makes a number of contributions. First, we contribute to the priming literature by providing evidence that when taxi drivers are primed with honesty, this increases the likelihood and intensity of fraudulent behavior. Thus we provide evidence from a natural field experiment in which an intended prime has led to unanticipated results or even ‘backfired’, complementing previous work where behavioral interventions had the opposite effect to that predicted (Lacetera & Macis, 2010; Mendoza et al., 2017; Muthukrishna et al., 2017), or results were unexpected (Kettle et al., 2017). Second, as the vast majority of the priming literature has taken place in the laboratory, we make a novel contribution by examining priming in a natural market setting devoid of intrusive experimenter scrutiny. Third, we contribute to the credence good literature by examining how making honesty, dishonesty and competition salient impacts actual charged prices, complementing previous field experiments that have examined how making price competition salient impacts quoted prices (List & Gneezy, 2014). Finally, we contribute to the literature that uses field experiments to examine how experimental manipulations can impact human behavior absent experimenter scrutiny.

The remainder of this paper is organized as follows. Section 2 describes the market place we study. Section 3 outlines our experimental design, motivates our treatments and outlines our hypotheses. Section 4 presents our results. In Section 5 we discuss our results, and Section 6 concludes.

## 2 THE MARKET

Vienna has about 4500 officially licensed taxis.^5^ There are thousands of passenger rides each week, hundreds of licensed taxi stands, and drivers can actively ply for hire by ‘cruising’ the streets or waiting at taxi stands. The market is highly regulated, and is comparable to taxi markets in other major cities such as London, New York and Athens.

As in other cities, regulation in Vienna means that taxis must use a tariff system that determines the price of a journey (the fare): for each journey, drivers charge passengers a fixed fee plus a distance and time dependent variable fee. The taxi meter is displayed prominently so the passenger can observe the price of the journey. Additionally, drivers can add extra charges conditional on the number of passengers, if the taxi was pre–ordered, if the journey destination is the airport, and any other special services.

Each extra charge can be added to the meter incredibly easily, either when the taxi has stopped, while the meter is still running, or both, and is done by pressing a small button on the front of the taxi meter. This button is typically located near to buttons that stop the journey, or change the variable fee. It is important to understand that for a driver to add any of additional charges to the fare, he must first consult with the passenger *by law*. Any charges that are added to the fare without the passenger's consent, or any wrongful manipulation of the variable fee is a form of overcharging and is illegal.^6^

## 3 EXPERIMENTAL DESIGN AND PROCEDURE

The experiment was designed to examine the effect of priming on the extent to which drivers fraudulently charge consumers in a real world market for credence goods. We use a field experiment to observe their behavior in a natural interaction without intrusive experimenter scrutiny. The subjects, the taxi drivers, were unaware that our study was taking place.

Although we have two primes that target overtreatment in their content (the *Dishonesty* and *Honesty* treatments), we measure both overcharging and overtreatment as there is a possibility that any prime might affect both. The previous literature on priming has shown that, in many cases, priming can have unexpected effects on behavior. For example, Bargh et al. (1996) find that priming college students with stereotypes about the elderly caused them to walk more slowly. Steele and Aronson (1995) find that African American students primed with their race underperform on academic tests, with similar impacts reported in other social groups (Aronson et al., 1998). Similar techniques have been used to examine risk and time preferences (Benjamin et al., 2010). We therefore measure both overcharging and overtreatment, to ensure our data–set is as rich as possible and that we do not overlook any potential behaviors.

### 3.1 Testers

To conduct the experiment we employed ‘testers’, or undercover confederates. The testers were hired through the subject pool at the Vienna Center for Experimental Economics, Austria. An advert was emailed to potential testers and stated that individuals were needed to assist researchers who were conducting a field experiment, the rate of pay was €10 per hour, and that this was a one time opportunity for employment. All testers were interviewed individually to guarantee sufficient English skills, were male, aged in their early to mid twenties, and wore casual clothing. Each tester was required to attend an hour long briefing and training session. The session gave testers the opportunity to practice the procedure, scripts and ask any questions. They received strict instructions about what they should say, being told to follow the scripts as closely as possible and not attempt to influence the driver in anyway. We selected only males in order to avoid any potential gender effects that might interact with our main treatment variables, as found by Castillo et al. (2013); Balafoutas et al. (2017); Grosskopf and Pearce (2020). Additionally only using male testers provides the best chance to avoid a ‘corner solution’ in cheating, as there is some evidence that women pay higher prices in credence goods markets (e.g., Alesina et al. (2013), Balafoutas et al. (2013)).^7^

### 3.2 Procedure

The experimental procedure we used is widely accepted in the literature (see Bertrand and Duflo (2017) and Baert (2018) for recent surveys of the audit method), and closely follows that of Balafoutas et al. (2013) and was conducted over weekdays in May 2018 (wave one) and February 2019 (wave two). At the beginning of each first day, testers were randomly assigned into groups of four. Each group was randomly assigned a sequence of 10 journeys, with all testers in a group completing the exact same sequence. For each journey in the sequence, all four testers took individual taxis from the same origin to the same destination, with a break of around 60 s between their journeys.^8^ All journeys began at taxi stands and ended at well known locations in the city.^9^ We refer to the four identical journeys taken in quick succession by a group of testers as a *quadruple*. The order in which testers caught taxis was randomized to control for potential order effects, and the small time difference between journeys within a quadruple rules out potential confounds, as the drivers would have faced identical driving conditions. In wave one, 20 testers completed a sequence of 10 journeys per day, and were employed for two days each. Wave one testers therefore completed 20 journeys each, and 400 journeys in total. In wave two, 20 additional testers completed a sequence of 10 journeys but were employed for one day only. Wave two therefore provides 200 journeys. This gives us a total of 40 testers, who completed a total of 600 journeys.

When taking a journey, every tester carried a GPS satellite logger that recorded the route the driver took, the distance they traveled and the amount of time taken to complete the journey. Testers also carried an experimental booklet, which they completed once the journey had ended. In the booklet, testers had to first record the driver's subjective appearance characteristics, recording age, gender, and ethnicity. Second, the testers were required to record detailed information about the taxi fare, distinguishing between the metered fare, the number of extra charges added by the driver, and the charged variable fare.^10^ Extra charges can be observed by watching the driver press buttons on the meter.^11^ With the exception of journeys taken to the airport, all of the journeys taken by our testers should have included no extra charges.^12^

Within a quadruple, each tester was randomly assigned to one of four treatments. For all treatments, the testers always entered the taxi at the front of queue, as is the norm in Vienna, and then spoke the following entry script, “I'd like to go to *destination x*. Do you know where it is? I am not from Vienna and I do not know the way,” where *destination x* is taken from the respective route of Tables A1–A3. This was spoken in English in order to signal to the driver that the passenger was a foreigner, and to ensure that all taxi journeys within the experiment would constitute a credence good (Balafoutas et al., 2013; Darby & Karni, 1973). This exact script is taken from Balafoutas et al. (2013), and was chosen because it represents a situation where the informational advantage of the seller is at its highest. The tester sat in the back of the taxi, as is common in Austria, and they were told that once they had spoken the script to act naturally, but distant, in order to avoid conversation.

The experimental treatments vary the prime that the testers spoke after the initial entry script. Each prime was spoken in English in order to maintain the signal that the passenger was a foreigner. Once spoken, the testers were instructed to sit in the back of the taxi in silence. Given the entry script, and the results from Balafoutas et al. (2013), any treatment effect of a prime in comparison to the *Baseline* is at a level at which fraud is already likely be reasonably high. We implemented four different treatments, the *Baseline*, *Honesty*, *Dishonesty* and *Uber* treatments. Table 1 summarizes the experimental design, and details each of the primes. The motivation for each treatment is given below. In each treatment, the information provided to drivers is truthful.

**TABLE 1:** Experimental design summary

Treatment	Entry script	Prime
Baseline	✓	No prime spoken.
Honesty	✓	“Did you hear about that study where researchers found that around 80% of taxi drivers were shown to behave honestly toward passengers, always taking them on the cheapest route? I read about it on the internet.”
Dishonesty	✓	“Did you hear about that study where researchers found that around 20% of taxi drivers were shown to behave dis‐honestly toward passengers, taking them on more expensive routes than necessary? I read about it on the internet.”
Uber	✓	“I checked the Über price online and it seemed cheap.”

We chose to use foreign testers for a number of reasons. First, following the credence good literature, we wanted to ensure that the taxi journeys we studied were in fact credence goods ‐ a foreign passenger signals the greatest informational asymmetry to the driver, and thus establishes that the ride is a credence good (as shown by Balafoutas et al., 2013). Using a local passenger could weaken this interpretation, as it is not clear that journeys would be viewed as credence goods by the drivers. Second, by speaking in a foreign language, the driver is given the opportunity to increase or reduce their fraudulent behavior in response to the primes. This is true along both the overtreatment and overcharging dimensions. If the passenger is local, there is little possibility that a driver can increase fraud especially if the passenger does not view the journey as a credence good. In addition, a driver would not be able to reduce fraud in response to the prime because he would not be committing fraud in the first place. Third, in our analysis we always compare any observed fraudulent behavior relative to other treatments. A consequence of this is that the effect of the tester being a foreigner, present in all treatments, should be netted out of any fraud we observe. Fourth, as English is the most common second language in many countries, the most common lingua franca in the world and the language most tourists are likely to use in an unknown foreign city, the results of our study are likely relevant to more people than if we had conducted the study in the local language (German).

Although data on taxi drivers' English skills is scarce, in the recent 2019 report by EF (an international education company) Austria ranks 8th and Vienna ranks in 6th place worldwide for English proficiency. Vienna is also a very popular tourist destination, and in 2018 had more than 1.5million English speaking tourists. Further, anecdotal evidence from correspondence with taxi companies suggests that the “majority” of drivers have good English skills.^13^

#### 3.2.1 Baseline treatment

The *Baseline* serves as a control treatment to which we can compare all others. No additional script was spoken in the *Baseline* treatment. As our testers all wore casual clothing, this treatment is similar to the *Foreigner Low Income* treatment reported in Balafoutas et al. (2013), but conducted in Vienna, Austria rather than in Athens, Greece, and with typical tourist destinations rather than low‐end hostels.

Although our *Baseline* treatment involves a particular statement, and emphasizes the knowledge difference between driver and passenger to a large extent, the script ensures that the knowledge difference is kept constant across journeys and treatments. This might encourage a high amount of overcharging, relative to previous studies. However, other studies also report high levels of overcharging, for example, Hall et al. (2019). Alternative ways of signaling this asymmetry could have been used ‐ for example, testers carrying luggage, carrying maps of the city or similar ‐ but these may be interpreted by the driver in ambiguous ways, which may in turn have then interacted with our treatments. The *Baseline* script ensures we maintain experimental control over the knowledge gap between driver and passenger, and ensures that the knowledge asymmetry is large.

An additional design consideration was whether to include a *Baseline* treatment with a local passenger or not. Doing so would have enabled us to identify the effect of information asymmetry on fraudulent behavior. Although this is an interesting line of inquiry, it has been previously studied in the taxi market albeit in Greece and not Austria (see Balafoutas et al. (2013)). We therefore chose not to include it for three reasons. First, although replication of experimental findings is important, the focus of our study is the effect of the primes on drivers' behavior, and multiple previous studies have examined local language passengers (Balafoutas et al., 2013, 2017). Second, the inclusion of a local *Baseline* treatment alone would not have been enough to identify the effect of the primes on driver behavior absent the foreign language effect: we would have had to implement three additional treatments in which the testers spoke the primes in the local language. This is due to possible interaction effects between primes and language that would not be identified if only the local *Baseline* was included. Operationally, this would have been difficult to implement as we would have to conduct *octuples* rather than *quadruples*, and we felt it more interesting to focus on the effects of the primes, rather than trying to identify the foreign language effect.

Finally, we decided to conduct the experiment in a foreign language (English) because many taxi drivers in Vienna are themselves not Austrian. Therefore, it is not clear that these drivers would be able to tell the origin of a passenger by their spoken German. This would add a source of ambiguity to our results.^14^ Although native German speakers can identify differences between (e.g.,) Germans, Austrians and Swiss Germans speaking German, for most other nationalities there are likely little to no differences. As many of our drivers are not Austrian natives, we felt that a German speaking baseline may have introduced heterogeneity in the drivers' beliefs about the origin of the passenger, and produce ambiguous responses to the treatments.

#### 3.2.2 Honesty and Dishonesty treatments

For the *Honesty* and *Dishonesty* treatments, testers spoke the exact same information to the drivers, however accentuate different aspects: the *Honesty* treatment emphasizes the positive portion of the information, stating that 80% of drivers were shown to behave honestly, whereas the *Dishonesty* treatment makes the negative salient, that is, by highlighting that 20% of drivers behaved dishonestly. We take these percentages from Balafoutas et al. (2013), who find that foreign passengers are defrauded in around 20% of the taxi journeys taken in Athens, Greece.^15^

The scripts for the *Honesty* and *Dishonesty* treatments are motivated by the following. First, although the seminal study by Balafoutas et al. (2013) focuses on the effect that information asymmetry has on both overtreatment and overcharging, the more recent study by Balafoutas et al. (2017) focuses mainly on overcharging. They find that, for non‐local passengers, ‘second–degree’ moral hazard influences overcharging behavior, but not overtreatment. As it has been previously shown that overcharging can be influenced through simple scripts, our scripts were designed to contribute to the literature by shedding light on how overtreatment could be manipulated. Second, various media articles suggest that the majority of the fraud committed by taxi drivers comes through the overtreatment channel, that is, by drivers taking extended detours. For example, a Czech taxi company writes on their website that, “[f]ortunately, the overcharging problem has got much better since the 1990s as a special taxi police team has been created to focus on this problem…[, t]oday, taxi scams in Prague are less frequent but despite that, many taxi drivers use other practices to legally inflate fares, for example, by using longer routes…”^16^ The *Honesty* and *Dishonesty* primes explicitly mention the route, the idea being to make overtreatment, or the possibility of route manipulation, salient.

Additionally these treatments are motivated by two strands of literature. First, they are conducted in the spirit of the priming literature (Liberman et al., 2004; Ward et al., 1997) and the literature examining valence framing effects (Levin et al., 1998), where casting the same information in a different light can produce large behavioral differences. Second, the primes are grounded in the idea that the majority of people view themselves as honest, and behave in line with some ethical code, but may need reminding to apply this code to their decisions (Kettle et al., 2017). We hypothesize that, by making honesty (dishonesty) more salient, the taxi drivers will behave more honestly (dishonestly) in comparison to the *Baseline*, and fraudulently treat customers by smaller (larger) amounts.

#### 3.2.3 Uber treatment

The *Uber* treatment is designed to prime drivers about their competitors and about price competition. However, the treatment is designed such that no actual pricing information is revealed, removing the possibility that the driver might form a reference point or target a particular fare. This treatment is motivated by Dulleck et al. (2011), who show that under certain competitive market conditions we would expect sellers to provide consumers with the appropriate quality of the good, but overcharge them. We hypothesize that priming sellers with competition will increase the extent to which they fraudulently overcharge consumers, relative to that observed in the *Baseline*.

## 4 RESULTS

In this section, we outline the results from the field experiment. We use a number of common features throughout the analysis. Where parametric and non–parametric tests are used, both the *p*–value and the test used are presented in parentheses. All tests are two sided unless otherwise stated. For all tests, the null hypothesis is always that there is no treatment difference. For parametric tests, the null hypothesis is always that the coefficient is equal to zero.

In total we collected 150 observations per treatment giving us a total of 600 observations.^17^ In all regression tables, as the number of explanatory variables is increased, the number of observations falls. This is due to missing entries in the data where driver characteristics were unable to be determined or recorded.

We first analyze if the treatments result in higher or lower fares relative to other journeys, and then decompose any effects into changes in overtreatment and overcharging.

### 4.1 Treatment effects

In order to determine if there exist any treatment effects, following Balafoutas et al. (2013), we first normalize the fares observed using prime *p* in quadruple *i*, *f*_*p*,*i*_, by dividing it by the fare of the cheapest journey from the same quadruple *i*, *f*_min,*i*_. The normalized fare, *F*_*p*,*i*_, for each prime *p*, for each quadruple *i*, is therefore calculated as

(1) $$F_{p,i}=\frac{f_{p,i}}{f_{\min,i}}.$$

The procedure that we employ for normalizing, as used in both Balafoutas et al. (2013) and Balafoutas et al. (2017), is based on the assumption that each journey, when considered individually, cannot reveal if the driver is overtreating the passenger or not. This is because the driver has expert knowledge about the road, conditions, fastest route and so on. Even if the driver deviates from a route deemed optimal by mapping software and takes a longer route, it is still not clear that the driver is overtreating the passenger: it may be that the driver has more information than the mapping software. This is because mapping software relies on user submissions regarding driving conditions, and is also not necessarily up‐to‐date regarding road layouts, and may omit information on road closures, traffic and crashes ‐ all variables that might impact a journey.^18^ However, two journeys taken at (almost) the same time, that both start from the same location, and go to the same destination are comparable as both contain the same natural shocks that impact a journey ‐ waiting at traffic lights, traffic conditions, construction work and road closures. Each journey provides the counterfactual to the other journey when identifying overtreatment. In our experiment, four journeys are taken at close intervals, which means driving conditions are orthogonal to the treatments assigned to each journey: all journeys within a quadruple, as they all start from the same location, and go to the same destination, all contain the same natural shocks that impact a journey. Using the minimum distance (or fare) within the quadruple as the counterfactual from which to compare the other journeys in the quadruple seems natural, as it is the journey with the least amount of overtreatment and therefore the one closest to the optimal journey. Normalization creates a clear and cogent index from which we can readily observe overtreatment, relative to the shortest distance. Table 2 provides a summary of the fare and normalized fare for each of the treatments.

**TABLE 2:** Summary statistics ‐ fares

	Baseline	Honesty	Dishonesty	Uber
Fare, €	15.51 (11.75)	15.68 (11.43)	15.36 (11.43)	15.69 (11.52)
Normalized fare	1.11 (0.16)	1.15 (0.24)	1.12 (0.18)	1.14 (0.27)
Observations	127	133	137	140

*Notes*: Normalized fares are calculated by dividing the paid fare from each treatment by the fare from the cheapest journey in each quadruple. Standard deviations in parentheses. Journeys where the driver has previously been observed are dropped from the analysis as are those where the driver did not complete the journey.

To formally examine if the fares are significantly different to the *Baseline*, we conduct a number of Tobit regressions. The marginal effects estimates from these regressions are presented in Table 3, models (1)‐(6). In each regression, the dependent variable is the normalized fare, and we include dummy variables that take values of 1 (and 0 otherwise) if the observation is from the *Honesty*, *Dishonesty* and *Uber* treatment. The *Baseline* treatment is always taken as the control. In regression (1), we do not include any controls in order to demonstrate the robustness of our estimates to the inclusion of covariates. Standard errors in each regression are clustered at the quadruple level.^19^

**TABLE 3:** Treatment effects on fares

Dependent variable	Normalized fare					
Model	(1)	(2)	(3)	(4)	(5)	(6)
Honesty treatment	0.044** (0.02)	0.046** (0.021)	0.048** (0.021)	0.053** (0.023)	0.059** (0.025)	0.055** (0.024)
Dishonesty treatment	0.011 (0.00)	0.009 (0.019)	0.008 (0.019)	0.012 (0.021)	0.001 (0.021)	−0.003 (0.021)
Uber treatment	0.029 (0.023)	0.035 (0.021)	0.036* (0.021)	0.041* (0.022)	0.031 (0.022)	0.038* (0.022)
Observations	537	482	482	449	449	449
Controls						
Set 1		✓	✓	✓	✓	✓
Set 2			✓	✓	✓	✓
Set 3				✓	✓	✓
Set 4					✓	✓
Set 5						✓

*Note*: *Baseline* treatment is taken as the baseline. Robust standard errors in parentheses, clustered by quadruple. Models (1)–(6) are Tobit regressions censored at 1. The presented explanatory variables are dummy variables that take a value of 1 if the observation is taken from that treatment (and 0 otherwise). ***, ** and * denote significance at the 1%, 5% and 10% level. All estimates are marginal effects.

We include five sets of controls, Set 1–5. In Set 1, we control for the day and timing of the journeys, whether or not the driver used a visible navigation system, a dummy variable controlling for the destination of the journey, and also controls for the driver's gender, age and ethnicity. We also control for the order in which the testers entered the taxis.^20^ We include a navigation system control because this may reveal to passengers if a driver goes on a detour, or that the driver is of a specific type. Destination specific controls are important in order to account for any signals that a destination might convey to drivers, thus influencing fraud. Set 2 includes a variable that controls for the average distance of the journeys within a quadruple. We include this in order to account for any quadruple specific effects associated with the distance that might otherwise be excluded. Set 3 controls for more quadruple specific variables, and includes the optimal distance as given by an online mapping software, and also the price an Uber would have cost for the same journey, collected at the time the journey took place. The Uber price is a variable that is correlated with journeys across a quadruple, and should take into account things such as traffic levels, construction, and other idiosyncrasies associated with journeys at the quadruple level. Set 4 includes tester specific dummy variables to account for tester specific effects. Set 5 adds dummy variables controlling for the origin of the journey. Thus, using these five sets of controls accounts for a comprehensive range of driver specific, journey specific and quadruple specific controls. In addition, we cluster standard errors at the quadruple level to account for within quadruple correlations.^21^

Observation 1. The Honesty treatment results in significantly higher fares.

*Support.* Table 3, models (1)‐(6) show that the coefficient estimate on the *Honesty* treatment dummy is positive and significant at the 5% level (*p* ≤ 0.05, in all cases, Wald Tests). Its magnitude is also robust to specification changes. The *Honesty* treatment produces fares that are between 4% and 5.5% larger than the *Baseline* fare in the quadruple.

Observation 1 outlines that fares are significantly larger in the *Honesty* treatment. However, from this observation alone it is not enough to determine if this is a result of overtreatment, or overcharging, or a possible combination of the two.

### 4.2 Overtreatment

To determine if Observation 1 is a result of overtreatment, we now examine the distance data collected by the GPS satellite loggers. If drivers are overtreating passengers in order to increase fares, we should observe this when comparing journey distances between treatments. To examine differences between treatments, we normalize distances in the same way that we normalize fares, dividing the observed distance, *D*, of prime *p* in quadruple *i*, by the shortest journey in the same quadruple, *D*_min,*i*_ providing a normalized distance measure *N*_*p*,*i*_

(2) $$N_{p,i}=\frac{D_{p,i}}{D_{\min,i}}.$$

Table 4 presents the average distances in each treatment in kilometres, and also the normalized distance. As can be seen, there exist only small difference between treatments. We formally examine if these differences are significant using Tobit regressions. The regressions are presented in Table 5, models (1)‐(6), and use the same sets of controls as those described in Section 4.1.

**TABLE 4:** Summary statistics ‐ journey distances

	Baseline	Honesty	Dishonesty	Uber
Distance, km	6.43 (5.91)	6.37 (6.02)	6.34 (5.97)	6.59 (6.12)
Normalized distance	1.1 (0.2)	1.1 (0.22)	1.11 (0.25)	1.13 (0.3)
Observations	127	133	137	140

*Note*: Normalized distances are calculated by dividing the distances from each treatment by the distances from the shortest journey in each quadruple. Standard deviations in parentheses. Journeys where the driver has previously been observed are dropped from the analysis as are those where the driver did not complete the journey.

**TABLE 5:** Treatment effects on distances

Dependent variable	Normalized distance					
Model	(1)	(2)	(3)	(4)	(5)	(6)
Honesty treatment	−0.009 (0.019)	0.003 (0.02)	0.007 (0.02)	0.008 (0.022)	0.007 (0.023)	0.005 (0.023)
Dishonesty treatment	0.003 (0.022)	0.002 (0.023)	0.001 (0.022)	0.003 (0.024)	−0 (0.024)	−0.001 (0.023)
Uber treatment	0.016 (0.021)	0.021 (0.024)	0.025 (0.024)	0.03 (0.026)	0.028 (0.026)	0.029 (0.025)
Observations	537	482	482	449	449	449
Controls						
Set 1		✓	✓	✓	✓	✓
Set 2			✓	✓	✓	✓
Set 3				✓	✓	✓
Set 4					✓	✓
Set 5						✓

*Note*: *Baseline* treatment is taken as the baseline. Robust standard errors in parentheses, clustered by quadruple. Models (1)–(6) are Tobit regressions censored at 1. The presented explanatory variables are dummy variables that take a value of 1 if the observation is taken from that treatment (and 0 otherwise). ***, ** and * denote significance at the 1%, 5% and 10% level. All estimates are marginal effects.

Observation 2. There are no treatment effects on journey distances.

*Support.* Table 4 shows that both distances and normalized distances are near identical across treatments. Table 5 formally supports this, with models (1)–(6) estimating the coefficient on all the treatment dummies to be close to zero and not significant at conventional levels (*p* > 0.1 in all cases, and in all models, Wald Tests).

Observation 2 suggests there is no difference in the level of overtreatment between the treatments. Importantly, Observation 2 does *not* imply that drivers are not overtreating our testers. On the contrary, it is potentially prevalent in all of our treatments as our *Baseline* treatment mimics the Balafoutas et al. (2013) *Foreigner Low Income* treatment to a large extent, and thus overtreatment might be already at a reasonably high level. We examine this further in Section 5.^22^ As fares are significantly higher in the *Honesty* treatment, but the level of overtreatment is identical across treatments, this suggests that drivers must be overcharging customers by larger amounts, or be overcharging them more frequently, in journeys in which they receive the *Honesty* prime.

### 4.3 Overcharging

We now examine the extent to which the differences between treatments can be explained by overcharging. We calculate overcharging by using an objective measure that is orthogonal to all treatments, and does not rely on our testers explicitly observing difficult to detect manipulations of the fare or the driver providing a fully itemized receipts. Unfortunately, unlike Balafoutas et al. (2013) who report that they received printed receipts for all their journeys, many drivers in Vienna hand wrote their receipts or produced printed receipts that did not itemize all the charges.^23^

As the GPS trackers records the distance of a journey, we can use this detailed information, along with the official taxi fare price list (see Appendix A2 and esp. Table A4 therein), to calculate the price a tester should have paid for a journey had no overcharging taken place. We call this the *Fair Price* of the journey. Importantly, this price includes any and all overtreatment committed by the driver. The *Fare*, which is the actual price the tester paid, is equal to the sum of the *Fair Price* and the amount of overcharging committed by the driver. Therefore, the difference between the *Fare* and the *Fair Price* must be equal to the amount the tester was overcharged. The amount of overcharging, *O*, observed when using prime *p* in quadruple *i* is therefore

(3) $$O_{p,i}=f_{p,i}-g_{p,i}.$$

where *g*_*p*,*i*_ is the *Fair Price* of prime *p* in quadruple *i*.

In order to compare the amount of overcharging between treatments in monetary amounts, we calculate the *Overcharging Difference* for each quadruple, calculating how much overcharging is observed for each journey using prime *p* in comparison to the journey with the smallest amount of overcharging in that quadruple *i*,

(4) $$R_{p,i}=O_{p,i}-O_{\min,i}.$$

where *O*_min,*i*_ is the journey in quadruple *i* with the smallest amount of overcharging.

**FIGURE 1:**
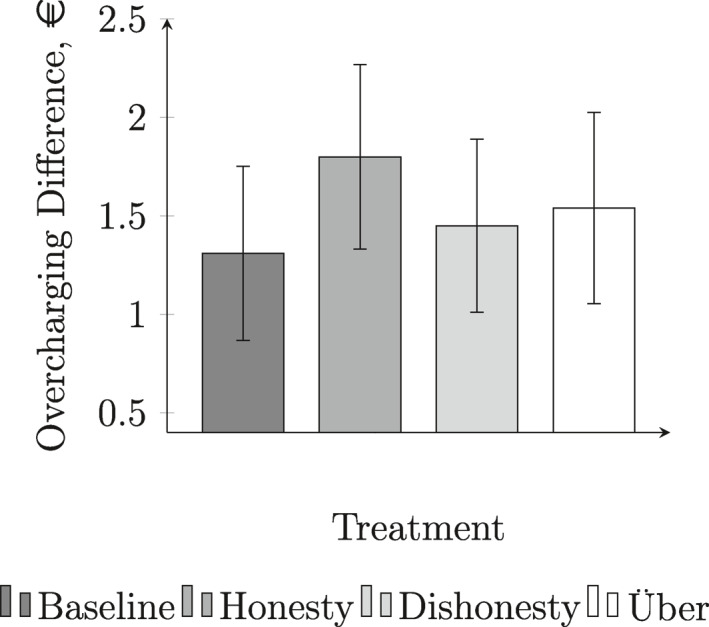
Overcharging Difference, €. Vertical bars represent 95% confidence intervals

Table 6 summarizes the proportion of journeys in which we observe a positive amount of overcharging, the average amount of overcharging, and also the average overcharging difference for each treatment. Figure 1 presents the overcharging differences graphically.

**TABLE 6:** Overcharging summary statistics

	Baseline	Honesty	Dishonesty	Uber
Proportion of journeys with overcharging	0.98 (0.15)	1 (0)	0.97 (0.17)	0.99 (0.08)
Total overcharging, €	5.31 (6.57)	5.69 (5.83)	5.3 (5.88)	5.41 (5.91)
Overcharging difference, €	1.29 (2.54)	1.77 (2.84)	1.45 (2.64)	1.55 (2.93)
Observations	127	133	137	140

*Note*: Standard deviations in parentheses.

We find that average levels of overcharging are comparable to that observed in the Balafoutas et al. (2013) *Foreigner* treatment, who report an average amount of overcharging conditional on it occurring of €5.99, with 22.4% of drivers overcharging something. In the Balafoutas et al. (2013) *Foreigner Low Income* treatment however, the average amount of overcharging, conditional on overcharging taking place is approximately €6.57, with 25.9% of all drivers overcharging something. We find that overcharging occurs in close to 100% of journeys, with the average amount of overcharging being around €5.60. Note, however, that a clean comparison of the studies is difficult, as the definitions of overcharging, the location of the studies, and the destinations of the rides (typical tourist places vs. low‐end hostels) vary. It appears as if drivers in our experiment use overcharging, rather than overtreatment, to fraudulently increase fares. We examine this further in Section 5.

To determine the effects of the primes on how much overcharging occurs, we conduct a number of Tobit regressions, with total overcharging as the dependent variable. In each regression, we include a dummy variable for each of the primes, and vary the same sets of controls as those described in Section 4.2. We use the same controls as those specified in Section 4.1, increasing the number used in each subsequent regression to examine the robustness of the estimates, and we cluster all observations at the quadruple level. Table 7 presents the estimates of the marginal effects.^24^

**TABLE 7:** Treatment effects on overcharging

Dependent variable	Overcharging difference					
Model	(1)	(2)	(3)	(4)	(5)	(6)
Honesty treatment	0.433** (0.201)	0.422** (0.207)	0.423** (0.205)	0.469** (0.216)	0.554** (0.221)	0.556** (0.22)
Dishonesty treatment	0.169 (0.201)	0.072 (0.181)	0.071 (0.184)	0.136 (0.194)	0.06 (0.186)	0.041 (0.176)
Uber treatment	0.238 (0.219)	0.294 (0.212)	0.295 (0.21)	0.373* (0.222)	0.281 (0.193)	0.35* (0.192)
Observations	537	482	482	449	449	449
Controls						
Set 1		✓	✓	✓	✓	✓
Set 2			✓	✓	✓	✓
Set 3				✓	✓	✓
Set 4					✓	✓
Set 5						✓

*Note*: *Baseline* treatment is taken as the baseline. Robust standard errors in parentheses, clustered by quadruple. Models (1)–(6) are Tobit regressions censored at 1. The presented explanatory variables are dummy variables that take a value of 1 if the observation is taken from that treatment (and 0 otherwise). ***, ** and * denote significance at the 1%, 5% and 10% level. All estimates are marginal effects.

Observation 3. Overcharging is greatest in the Honesty treatment.

*Support*. Table 7, models (1)‐(6) outline how the coefficient on the *Honesty* priming dummy is always positive and significant (*p* < 0.05). The magnitude and sign of the coefficient is robust to specification changes. This suggests the *Honesty* prime significantly increases the amount of overcharging by a driver relative to the *Baseline* treatment.

Observation 3 highlights how the *Honesty* treatment increases fares by increasing the intensity overcharging. This observation goes against our initial hypothesis that the *Honesty* prime would reduce fraudulent behavior. It is also interesting to note that the *Dishonesty* treatment has no discernible effect on the behavior of drivers, despite the exact same information being conveyed to the driver. The coefficient is always found to be not significantly different from zero. The *Uber* treatment, although significant at the 10% level in model (3) and (5), is not significant at more conventional levels and is not estimated to be significant in any other regression.

## 5 DISCUSSION

Our observations raise the following questions: what can explain the treatment effects we observe? Why do drivers use overcharging instead of overtreatment? To what extent can our observations be explained by informational asymmetries between seller and buyer? We examine these questions in the following two subsections. We discuss potential explanations in 5.1, and additional analyses in Section 5.2 in order to shed light on the role played by knowledge asymmetries.

### 5.1 Potential explanations

A compelling explanation for why drivers use overcharging rather than overtreatment is that it is the easiest and most efficient way to defraud passengers ‐ and the most difficult to spot. Even our testers, who had been trained to observe drivers attempting to cheat them by overcharging, acknowledged that there were instances where they may have missed drivers doing so. This is one reason why we use the measure of overcharging that we do. In contrast, overtreatment requires drivers to take detours that may be difficult to plan. This is particularly true in old European cities where many roads are rarely straight, often only two lanes wide with cars parked down both sides, and grid plans (common in ‘new’ cities, especially in the US) are not in use. It may also be that, with the increased use of mobile phones with GPS satellite navigation, overtreatment is more easily detected by passengers.

In explaining the results from the Uber treatment, we acknowledge it has a number of limitations. First, because we do not quote a specific price to drivers, drivers' perceptions of what constitutes a ‘cheap’ price is ambiguous: drivers may not be aware of Uber's pricing, especially as they use variable pricing. Thus, stating that Uber's price ‘seemed cheap’ may have had a heterogeneous effect on the drivers behavior. It is conceivable that drivers charged a higher price to those they perceived as having a higher reference point, and this may not be constant across drivers. Second, it is possible that because our testers spoke English, drivers may have been unaware of where they are from, and the prices they are using as a comparison. This too may have had a heterogeneous effect on drivers, and can explain why we find such a weak treatment effect.^25^

An explanation for the drivers' response to the *Honesty* treatment could be related to their beliefs about the proportion of drivers behaving honestly. As the prime emphasizes the proportion of drivers behaving honestly, upon hearing the prime the driver may update his beliefs such that he believes the passenger believes the majority of drivers behave honestly, and that the customer is trusting taxi drivers in general. As the truthful information provided to drivers was taken from a study conducted in Athens, rather than Vienna, it is possible the information altered their beliefs significantly. The driver's belief about the probability that he would be scrutinized by the passenger would then go down, and be smaller than if the *Honesty* prime were not spoken, that is, would be smaller than beliefs in the *Baseline*. As such, drivers hearing the *Honesty* prime would have a greater incentive to defraud the passenger in comparison to the *Baseline*. An alternative explanation for the overcharging result we observe in the *Honesty* treatment is that, as the prime focuses on overtreatment but excludes overcharging, the driver now believes that the passenger defines an honest behavior as taking the cheapest route ‐ which makes overcharging permissible.

In contrast, the *Dishonesty* treatment emphasizes the proportion of dishonest drivers. Upon hearing the prime, a driver may form a belief that the passenger believes a large proportion of drivers behave dishonestly, and are therefore *more* likely to inspect his behavior, that the customer is suspicious of taxi drivers, and if the taxi driver wants to defraud the customer, he has to be more careful. This may then cause the driver to reduce the level of fraud, in comparison to the *Baseline*. However, as the experimental design is such that the informational asymmetry between the passenger and driver is high, drivers may believe there is a minimum level of fraud that they can successfully apply to the fare without being caught. This would then explain why drivers do not reduce the amount of fraud in the *Dishonesty* treatment in comparison to the *Baseline*. Our results are consistent with all these explanations, but we acknowledge that we are unable to distinguish between them.

### 5.2 The role of asymmetry in knowledge

Although we do not have information about how ‘local’ informed passengers might be treated by the drivers in our sample, we do have information about how a perfectly informed passenger would be treated. We also have evidence from mystery shopper journeys that local passengers are not treated fraudulently. Two survey institutes in Austria regularly conducted ‘mystery shopper’ taxi journeys in Vienna, latest in 2017, and 2019. Each study was conducted using similar procedures to that employed in our present study. Testers took predetermined taxi journeys and compared them to an optimal route. The testers in both studies were locals, and spoke to the driver in German. Both studies find, from a combined total of 1245 journeys, that the vast majority of taxi drivers (1) take the optimal route and (2) there is no evidence that they overcharge passengers.^26^ We take this as evidence that both overtreatment and overcharging rates are small to non‐existent at the local level.

During the study, we collected (1) the ‘optimal’ route for each journey planned using Google Maps and (2) the price of each journey had an Uber taxi been taken instead. The Google Maps distances provide an estimate of the journey a driver would take if he was aware the passenger was informed. The Uber prices were collected during the experiment, prior to each journey taking place, and are provided by an algorithm in the Uber software that gives an ‘estimated’ price of a particular journey, and assumes the optimal route is taken by the driver. This price includes wait times, and incorporates traffic information. In addition, we also collected a third set of information, (3) the distance of each journey planned using Open Street Maps (OSM, hereafter).

The three sets of distance and price information can be used to provide alternative baselines from which we can compare the drivers' behavior in our treatments. Specifically, we can compare the observed fares from our experiment to the fares calculated using the Google Maps and OSM distances and taxi fare tables in order to examine the extent to which our testers were fraudulently charged relative to a perfectly informed passenger. Taking each observed fare using prime *p* in quadruple *i*, *f*_*p*,*i*_ and dividing it by the fare calculated using the Google Maps distance, *f*_*google*,*i*_, the fare calculated using the OSM distance, *f*_*OSM*,*i*_, or the Uber price, *f*_*uber*,*i*_, associated with each quadruple *i*, provides us with three additional normalized fares:

(5) $$F_{p,i,k}=\frac{f_{p,i}}{f_{k,i}}$$

where *k* ∈ {*Google Maps*, *OSM*, *Uber*}. Table 8 presents the normalized fares using each of the three additional prices.

**TABLE 8:** Normalized fares ‐ Informed passenger baselines

	Baseline	Honesty	Dishonesty	Uber
Normalized fare, Google Maps price	1.4 (0.3)	1.43 (0.33)	1.41 (0.28)	1.43 (0.38)
Normalized fare, Open Street Maps price	1.39 (0.29)	1.44 (0.33)	1.41 (0.29)	1.44 (0.38)
Normalized fare, Uber price	1.38 (0.42)	1.45 (0.47)	1.41 (0.4)	1.45 (0.5)
Observations	127	133	137	140

*Note*: Normalized fares are calculated by dividing the paid fare from each treatment by the fare associated with the Google Maps, OSM distance or the estimated price provided by Uber. Standard deviations in parentheses.

As can be seen, the fares in each of our treatments are more than 40% larger than the Google Maps, OSM and Uber prices on average, with only small variations across treatments and across each of the three baselines. This suggests that there is a significant amount of fraud, relative to a perfectly informed customer across treatments, no matter the measure of ‘perfectly informed’ that we use. As the Uber price was collected during the experiment, and as the normalized fares using the Uber price are very similar to both the Google Maps and OSM normalized fares, we take this as evidence for their suitability for use to normalize distances.

We estimate similar regressions as those in Table 3 in order to determine any treatment effects when using these alternative baselines. The dependent variable is the normalized fare, as given in Equation (5). We present the results in Table 9, providing the marginal effect estimates from the Tobit regressions for each of the treatment dummies. In addition, the estimates of the constants from the main regressions are also provided.^27^

**TABLE 9:** Treatment effects on fares—Google baseline

Dependent variable	Normalized fare					
Model	(1)	(2)	(3)	(4)	(5)	(6)
Honesty treatment	0.034 (0.025)	0.044 (0.027)	0.043 (0.027)	0.049* (0.029)	0.052* (0.031)	0.048 (0.031)
Dishonesty treatment	0.009 (0.023)	−0.01 (0.025)	−0.009 (0.025)	−0.005 (0.025)	−0.015 (0.028)	−0.016 (0.026)
Uber treatment	0.034 (0.03)	0.037 (0.029)	0.037 (0.03)	0.04 (0.031)	0.037 (0.032)	0.054* (0.032)
Constant	1.397*** (0.263)	1.873*** (0.154)	1.853*** (1.574)	1.849*** (0.173)	1.87*** (0.186)	1.17*** (0.242)
Observations	537	482	482	449	449	449
Controls						
Set 1		✓	✓	✓	✓	✓
Set 2			✓	✓	✓	✓
Set 3				✓	✓	✓
Set 4					✓	✓
Set 5						✓

*Note*: *Baseline* treatment is taken as the baseline. Robust standard errors in parentheses, clustered by quadruple. Models (1)–(6) are Tobit regressions censored at 1. The presented explanatory variables are dummy variables that take a value of 1 if the observation is taken from that treatment (and 0 otherwise). ***, ** and * denote significance at the 1%, 5% and 10% level. All estimates are marginal effects, except for the constant which is taken from the main Tobit regression.

Observation 4. All treatments produce significantly higher fares than the perfectly informed passenger baseline.

*Support.* As can be seen from Table 9, the constant in all regressions is estimated to be large, and significant at the 1% level. This implies that *all* treatments produce a large and significant (*p* < 0.01 in all cases, Wald Tests) increase in fares in comparison to the perfectly informed passenger baseline. The constant is significantly different to 1 in all cases (*p* < 0.001 in all cases, Wald Tests). However, there are no significant differences in the fare increases between the treatments at conventional levels (*p* > 0.05 in all cases, Wald Tests).

We can now decompose what might be driving these increases in fares. Using the distances from the route mapping software to produce normalized distances, we can examine the extent to which our testers' were overtreated. Each normalized distance is calculated as follows,

(6) $$N_{p,i,j}=\frac{D_{p,i}}{D_{j,i}}$$

where *D*_*p*,*i*_ is the observed journey distance of prime *p* in quadruple *i*, and *D*_*j*,*i*_ the distance produced by the mapping software for the journey in quadruple *i*, where *j* = {*Google Maps*, *OSM*}.

Table 10 presents the normalized distances, using the Google Maps and OSM as baselines. As can be seen, average normalized distances are close to one in all cases, and not significantly different to 1 (*p* > 0.1 in all cases, Sign Test), suggesting there is no overtreatment in comparison to the optimal routes. Our testers are taken on routes that are on average indistinguishable from those a driver would take were they a perfectly informed passenger. Although these hypothetical routes are arguably not ‘perfect’ counterfactuals, this provides some support that the Google and OSM distances are suitable for use as a robustness check.

To examine this further, Table 11 presents the marginal effects estimates from a number of Tobit regressions, using the normalized distance with a Google Maps baseline as the dependent variable, and the same sets of controls as those in Section 4.^28^

As can be seen, the coefficients on the dummy variables for each of the treatment variables are not significantly different to zero at conventional levels (*p* > 0.1, in all cases, Wald Tests). The constant when no controls are included is also very close to 1, and only significantly different in model (4) (*p* > 0.1 in all models except (4), *p* = 0.04 in model (4), F–tests). We take this as evidence that journeys are close to the hypothetical optimal. It suggests asymmetry in knowledge is not driving overtreatment. Interestingly, this is consistent with evidence supplied to us by the official local representative of the Viennese Taxi drivers (from the Federal Chamber of Economy Austria, WKO) who wrote to us to explain that the overtreatment of foreign passengers happens in very few cases only. Whenever the representative is informed about misconduct (a criminal offense) the police are contacted. In addition this result is also consistent with the evidence from the ‘mystery shopping taxi experiments’ conducted by the previously mentioned survey institutes. As we have shown, our foreign passengers are indeed taken on the optimal routes, and there is no difference compared to fully informed (local) passengers. As we observe no overtreatment relative to the optimal journey, this implies that the increased fares relative to the perfectly informed passenger baselines must be a consequence of overcharging. We add this analysis to Appendix A4, which confirms that overcharging is driving the differences between our observations and the optimal journey.

One additional point to mention is that a foreign passenger and a perfectly informed passenger face different types of goods. While a taxi ride constitutes a credence good for a foreigner (see e.g., Balafoutas et al. (2013)) it can be interpreted as an ordinary good for locals. Thus any price differences are credence goods markups (see Hall et al. (2019) for a motivation and the terminology). Interestingly Hall et al. (2019) report a credence good markup of nearly the same magnitude, as the one in our study reported in Table 8, namely about 40%.

That we report limited evidence of overtreatment, but significant levels of overcharging in all treatments is intriguing, particularly when considering the levels of overtreatment reported by Balafoutas et al. (2013). One potential explanation for this is that, over the past 10 years, satellite navigation systems on the mobile phones of passengers have become ubiquitous, and passengers may have better (or perfect) information about optimal routes. However, rather than eliminating fraud, it instead ‘shifts’ it to another ‘less verifiable’ dimension of the service.

**TABLE 10:** Overtreatment ‐ Informed passenger baselines

	Baseline	Honesty	Dishonesty	Uber
Normalized distance, Google Maps	1.01 (0.25)	0.99 (0.21)	1 (0.25)	1.01 (0.25)
Normalized distance, Open Street Maps	1 (0.24)	0.98 (0.22)	0.98 (0.26)	1.01 (0.25)
Observations	127	133	137	140

*Note*: Normalized fares (distance) are calculated by dividing the paid fare (distance) from each treatment by the fare (distance) from the shortest journey in each quadruple. Standard deviations in parentheses. Some journeys from the *Honesty*, *Dishonesty* and *Uber* treatments were dropped, due to them being repeated observations of the same driver.

**TABLE 11:** Treatment Effects on Distances ‐ Google baseline

Dependent Variable:	Normalized Distance, Google Maps					
Model	(1)	(2)	(3)	(4)	(5)	(6)
Honesty treatment	−0.022 (0.014)	−0.017 (0.015)	−0.017 (0.015)	−0.017 (0.015)	−0.016 (0.018)	−0.016 (0.017)
Dishonesty treatment	−0.01 (0.00)	−0.015 (0.016)	−0.019 (0.015)	−0.021 (0.016)	−0.02 (0.017)	−0.024 (0.016)
Uber treatment	−0.002 (0.015)	−0.003 (0.016)	−0.003 (0.016)	−0.004 (0.017)	−0.003 (0.019)	−0.01 (0.017)
Constant	0.921*** (0.345)	1.082*** (0.163)	1.293*** (0.152)	1.287*** (0.135)	1.204*** (0.162)	1.322*** (0.233)
Observations	537	482	482	449	449	449
Controls						
Set 1		✓	✓	✓	✓	✓
Set 2			✓	✓	✓	✓
Set 3				✓	✓	✓
Set 4					✓	✓
Set 5						✓

*Note*: Robust standard errors in parentheses, clustered by quadruple. Models (1)–(6) are Tobit regressions censored at 1. The presented explanatory variables are dummy variables that take a value of 1 if the observation is taken from that treatment (and 0 otherwise). ***, ** and * denote significance at the 1%, 5% and 10% level. All estimates are marginal effects, except for the constant which is taken from the main Tobit regression.

## 6 CONCLUSION

We present a natural field experiment designed to examine if priming can reduce the fraudulent behavior of taxi drivers in a real world market for credence goods. Using undercover passengers equipped with GPS satellite loggers, we collected data on 600 individual taxi rides in the Austrian capital of Vienna. Using a 4 × 1 between–subject design, we exogenously varied the prime spoken to drivers. Building on the novel experimental design of Balafoutas et al. (2013), we minimize potential confounds by taking journeys in *quadruples*, each within 60 s of each other. The data gained from the GPS trackers along with the comprehensive data collected by the testers, and from receipts, enables us to distinguish between the two channels of fraud in this market: overtreatment and overcharging. In contrast to our hypotheses, our main conclusion is that the priming either increased or had no effect on the fraudulent behavior of taxi drivers. While the *Honesty* treatment is shown to increase total fares by around 5.5%, the *Dishonesty* and *Uber* primes does not influence prices. Analyzing the channels through which prices could be inflated, we find no evidence that the primes increase overtreatment. Instead, we report drivers overcharging in the *Honesty* treatment.^29^

We acknowledge that we have focused on a single, one–shot interaction and cannot comment on any long–term impacts that our primes may have on taxi drivers. In particular, we cannot shed light on how the same driver faced with repeated primes might behave, or how the same driver primed with two different primes in sequential journeys might respond. Further, we cannot address how the driver might behave toward subsequent passengers if they too used a prime, or even if they used no prime at all. However, we have shown that using priming as a low cost tool to reduce fraud in markets for credence goods is detrimental for the consumer. Although the informational asymmetry between the expert seller and the customer is kept constant, our primes increases the price charged by the expert. The main implication of this is that a consumer who finds herself to have an informational disadvantage in comparison to a seller should say as little as possible, reducing the possibility of increasing dishonest behavior further.

As we find that our primes had the opposite effect to that which we had anticipated, our study highlights how interventions predicted to produce one result can often ‘backfire’ or produce unexpected effects. A number of studies examining both priming and other behavioral interventions have reported similar results (Lacetera & Macis, 2010; Mendoza et al., 2017; Muthukrishna et al., 2017). In general, and in–line with the suggestions of Levitt and List (2007), further examinations of priming in the field will shed light on both their effectiveness in different contexts and the extent to which the results obtained from the laboratory generalize to other settings. Future investigations of the stability of priming effects across time might prove fruitful. Other interesting avenues for further research could be to focus on primes that have previously been found to affect behavior in the lab, and if primes can effect other economically relevant behaviors.

## Supporting information

Supplementary Material 1

Supplementary Material 2

## ENDNOTES

^1^
Balafoutas et al. (2017) define ‘second degree’ moral hazard as, “the tendency of the supply side in a market to react to anticipated moral hazard on the demand side by increasing the extent or price of the service”. In a field experiment, they find that taxi drivers increase charges when a buyer tells them they will ask their employer to reimburse the taxi receipt, in comparison to when they do not.

^2^
See Kerschbamer and Sutter (2017) and Balafoutas and Kerschbamer (2020) for recent reviews of lab and field experiments in credence goods markets.

^3^
We refer to Cohn and Maréchal (2016) for a review of priming in experimental economics.

^4^
Related work in psychology also highlights how unethical actions can also lead to individuals forgetting moral rules (Shu & Gino, 2012).

^5^
See https://wien.orf.at/v2/news/stories/2921228/, accessed latest on 30/07/2020, for more details.

^6^
The full tariff system, and a list of extra charges that could be added, are given in Appendix A2, Table A4.

^7^
We use a relatively large number of testers for the following reasons. First, as previous studies have criticized the audit methodology on the grounds of testers finding what they believe the experimenter wants to find (Heckman, 1998), we used a large number of testers in order to reduce the influence that any one tester might have on the results. Second, having a small number of testers conduct the experiment over many days, or weeks, taking hundreds of rides each, might allow them to more easily guess the experimental hypothesis which may have resulted in them altering their behavior. Finally, a small number of testers would make them more easily recognisable to drivers, which may have raised suspicions.

^8^
As far as we are aware, no drivers suspected that a study was taking place. The city of Vienna has thousands of taxi drivers, and taxi stands are very busy in general, so it seems reasonable that drivers would not expect anything strange seeing four passengers take taxis in 4 minutes. In many cases, there are long queues of taxis and many passengers take journeys in between our testers within a quadruple. Keeping taxi journeys close together in time also ensures that journeys within a quadruple are comparable, as highlighted by Balafoutas et al. (2013).

^9^
Tables A1–A3 provides the origins and destinations of all the journeys taken.

^10^
Testers were at no point informed about the purpose or hypotheses of the study.

^11^
Although all drivers in Vienna are required by law to provide a printed receipt that distinguishes between the metered fare and any extra charges, some drivers still provide hand written receipts without sufficient information. Others provide printed receipts that do not distinguish between charges, or lack other information.

^12^
Drivers are allowed to include an extra charge of €13 for journeys to the airport.

^13^
As part of the study, we asked our testers to judge if they felt the drivers understood what was spoken to them in English. Testers were asked to be conservative in their judgment, and report either yes or no. In 80% of journeys, testers believed the driver understood everything spoken to them, and there are no significant differences between treatments. All the results presented in Section 4 are robust to controlling for English understanding as reported by our testers.

^14^
In our sample, less than 20% of drivers were reported to be Austrian. A newspaper article reports that the majority of Viennese drivers has immigrant background, coming from around 50 different ethnic groups. Table A5 in the Appendix outlines the nationalities of the drivers of our experiment, and the article can be found here https://www.wienerzeitung.at/nachrichten/chronik/wien/403925‐Privatdetektive‐testen‐Taxifahrer.html, accessed latest on 30/07/2020.

^15^
Although we have no evidence that the drivers felt themselves as part of a study, there are infrequent ‘mystery shopping’ taxi experiments conducted by Viennese survey institutes. It is important to note that had drivers thought they were part of a study or that they were carrying a mystery shopper, it would have been best for them to not defraud the tester at all ‐ avoiding any potential disciplinary action or problems. Thus, our results could be interpreted as a lower bound on fraudulent behavior.

^16^
See https://www.prague‐airport‐transfers.co.uk/other‐transport/extras‐and‐special‐requests/dishonest‐drivers‐taxi‐service‐in‐prague/ for more information, accessed latest on 30/07/2020.

^17^
Due to the unique license plate being written on the majority of receipts we have been able to check whether we accidentally caught a driver twice. Note that 12 observations in each of the *(Dis)Honesty* treatments and 8 observations in the *Uber* treatment had to be excluded from the analysis for this reason. 19 are dropped from the *Baseline*, leaving us with a total of 549 observations. As we only examine foreign passenger journeys, we acknowledge that our experimental results cannot be generalized to journeys with local passengers. In Section 5 we provide some discussion of the results, along with some additional analysis. Our results are quantitatively similar if we include repeated driver observations.

^18^
These reasons highlight why fares and distances calculated from mapping software are arguably not a reasonable counterfactual from which to draw the main body of our conclusions relating to treatment effects.

^19^
Throughout the paper, if we estimate the marginal effects with all observations ‐ including those where the journey was not completed, and where we had repeated observations of the same driver ‐ the results are near identical.

^20^
In four journeys, the driver is recorded as being unable to complete the journey. Anecdotal evidence suggests that this has been due to construction work preventing the driver completing the journey. An additional eight cannot be verified as being completed or not. All 12 observations are dropped from the analysis.

^21^
All our observations and estimates are robust to clustering standard errors ‐ estimates are near identical if standard errors are not clustered.

^22^
It is important to note that, by looking at the logged GPS data, there is limited evidence that the vast majority of drivers took different routes to get to the destination. However, some drivers quite clearly do take extended journeys. The raw GPS data is available from the authors upon request.

^23^
Although this is illegal in Austria, it appears to be a common occurrence, something we had not anticipated before we conducted the study.

^24^
The results without clustering at the journey level are quantitatively similar and available upon request.

^25^
Unfortunately, the Uber treatment sample size is too small for a meaningful exploration of the possible heterogeneity.

^26^
We thank the principal of the ‘mystery shopping experiments’, ‘Taxi 40100’ for their cooperation in answering a set of questions.

^27^
The results from the alternative baselines are near identical. Only the Google Maps baseline results are presented for brevity, with others relegated to Appendix A4.

^28^
Again, for brevity, we relegate estimates using the OSM baseline to the Appendix. For both the OSM and Uber alternative baselines, the results are near identical to those presented in Table 10.

^29^
It is important to understand that although our results pertain to three particular priming instruments, it is not necessarily clear how priming honesty, dishonesty and competition in alternative ways might influence behavior. Despite this, our results still shed light on how an uninformed customer might be treated by an informed expert and how priming can influence the expert's behavior.
