# Supplementary material for: The effect of priming on fraud: Evidence from a natural field experiment
Source: Econ Inq. 2022 Apr 30;60(4):1854–74. doi: 10.1111/ecin.13088 (PMC9540625; doi:10.1111/ecin.13088)
Supplement: Supplementary file 1 — Supplementary Material 1 [file ECIN-60-1854-s002.pdf]

ORIGINAL ARTICLE DISCLOSURE STATEMENT

WEAI

This form must be submitted with your manuscript before it will enter the review process. Answer 'none' if such is the case. If accepted for publication, a PDF of this document will be posted online with your article. Additionally, include any necessary funding information and acknowledgements you wish to see in print in your manuscript document file.

Manuscript Title	The effect of priming on fraud: Evidence from a natural field experiment
Submitted To	☐ *Contemporary Economic Policy* ☒ *Economic Inquiry*

Each author must disclose if another party had the right to review the paper prior to its circulation.
None
Each author must disclose any paid or unpaid positions that they hold (or any close relative or partner holds) as officer, director, or board member of relevant non-profit organizations, or profit-making entities. A relevant organization is one whose policy positions, goals, or financial interests relate to the article.
None
Identify each interested party from whom you (or any close relative or partner) have received financial support of at least $10,000 in the past three years, in the form of consultant fees, retainers, grants, and the like (including in-kind support, such as providing access to data). If the support in question comes with a non-disclosure obligation, that fact should be stated, along with as much information as the obligation permits. An "interested" party is any individual, group, or organization that has a financial ideological, or political stake related to the article.
None
For any paper involving the collection of data on human subjects the author(s) must disclose whether they have obtained Institutional Review Board (IRB) approval; if no IRB approval was obtained, the reason should be stated.
IRB by the University of Innsbruck (SFB F63)

Signature: [signature] Date: 8.6.2021

Name (printed): PARAMPREET CHRISTOPHER BINDRA

ORIGINAL ARTICLE DISCLOSURE STATEMENT

WEAI

This form must be submitted with your manuscript before it will enter the review process. Answer 'none' if such is the case. If accepted for publication, a PDF of this document will be posted online with your article. Additionally, include any necessary funding information and acknowledgements you wish to see in print in your manuscript document file.

Manuscript Title	The effect of priming on fraud: evidence from a natural field experiment
Submitted To	☐ *Contemporary Economic Policy* ☑ *Economic Inquiry*

Each author must disclose if another party had the right to review the paper prior to its circulation.
N/A
Each author must disclose any paid or unpaid positions that they hold (or any close relative or partner holds) as officer, director, or board member of relevant non-profit organizations, or profit-making entities. A relevant organization is one whose policy positions, goals, or financial interests relate to the article.
N/A
Identify each interested party from whom you (or any close relative or partner) have received financial support of at least $10,000 in the past three years, in the form of consultant fees, retainers, grants, and the like (including in-kind support, such as providing access to data). If the support in question comes with a non-disclosure obligation, that fact should be stated, along with as much information as the obligation permits. An "interested" party is any individual, group, or organization that has a financial ideological, or political stake related to the article.
N/A
For any paper involving the collection of data on human subjects the author(s) must disclose whether they have obtained Institutional Review Board (IRB) approval; if no IRB approval was obtained, the reason should be stated.
IRB approval obtained from the University of Innsbruck, SFB F63

Signature: [signature] Date: 6/1/22

Name (printed): Graeme Pearce
