# Supplementary material for: The effect of priming on fraud: Evidence from a natural field experiment
Source: Econ Inq. 2022 Apr 30;60(4):1854–74. doi: 10.1111/ecin.13088 (PMC9540625; doi:10.1111/ecin.13088)
Supplement: Supplementary file 2 — Supplementary Material 2 [file ECIN-60-1854-s001.pdf]

Online Appendix to The effect of priming on fraud: evidence from a natural field experiment

Appendix - For online publication

A1 Routes

Route No.	*Origin*	*Destination*	*Uber Price*
1	Main Station	Wiener Staatsoper	7.5
2	Hotel Sacher	Schottentor	10.5
3	Schottentor	Messe Wien	14.5
4	Messe Wien	Vienna Marriott	8
5	Vienna Marriott	Alser Spitz	10.5
6	Alser Spitz	Main Station	9.5
7	Main Station	Sports Center Gudrun	13
8	Sports Center Gudrun	Vienna Westbahnhof	7
9	Vienna Westbahnhof	Vienna Airport Arrival	31
10	Vienna Airport Arrival	Main Station	10
11	Main Station	Zipperstreet Underground Station	14.5
12	Zipperstreet Underground Station	Meidling Station	13.5
13	Meidling Station	U4 Vienna	10
14	U4 Vienna	Hietzing Hospital	18
15	Hietzing Hospital	Kennedy Bridge	11.5
16	Kennedy Bridge	Johann-Nepomuk-Berger-Place	5.5

Table A1: Routes Part 1/3

Route No.	*Origin*	*Destination*	*Uber Price*
17	Johann-Nepomuk-Berger-Place	Nussdorfer Street Station	7
18	Nussdorfer Street Station	Sieveringer Street McDonalds	6
19	Sieveringer Street McDonalds	Alterlaa Bowling Center	28.5
20	Alterlaa Bowling Center	Main Station	17.5
21	Main Station	Vienna Meidling Station	7.5
22	Vienna Meidling Station	Alterlaa Bowling Center	12.5
23	Alterlaa Bowling Center	Vienna Liesing Station	8.5
24	Vienna Liesing Station	Vienna Uno city	30.5
25	Vienna Uno city	Rennbahnweg Station	9
26	Rennbahnweg Station	Messe Wien	12
27	Messe Wien	Schottentor	11.5
28	Schottentor	Ambassador Hotel	9.5
29	Ambassador Hotel	Vienna Marriott	5
30	Vienna Marriott	Main Station	7
31	Main Station	Vienna Airport Arrival	30
32	Vienna Airport Arrival	Vienna Westbahnhof	32

Table A2: Routes Part 2/3

Route No.	*Origin*	*Destination*	*Uber Price*
33	Vienna Westbahnhof	Sports Center Gudrun	11.5
34	Sports Center Gudrun	Main Station	6.5
35	Main Station	Alser Spitz	10.5
36	Alser Spitz	Vienna Marriott	8
37	Vienna Marriott	Messe Wien	10
38	Messe Wien	Schottentor	12
39	Schottentor	Wiener Staatsoper	7.5
40	Hotel Sacher	Main Station	6.5
41	Main Station	Alterlaa Bowling Center	10
42	Alterlaa Bowling Center	Sieveringer Street McDonalds	35.5
43	Sieveringer Street McDonalds	Nussdorfer Street Station	6
44	Nussdorfer Street Station	Johann-Nepomuk-Berger-Place	8
45	Johann-Nepomuk-Berger-Place	Kennedy Bridge	9
46	Kennedy Bridge	Hietzing Hospital	7.5
47	Hietzing Hospital	U4 Vienna	11
48	U4 Vienna	Meidling Station	4
49	Meidling Station	Zipperstreet Underground Station	7.5
50	Zipperstreet Underground Station	Main Station	9

Table A3: Routes Part 3/3

A2 Vienna taxi tariffs

The Viennese taxi fares are taken from https://www.wko.at/branchen/w/transport-verkehr/befoerderungsgewerbe-personenkraftwagen/Wiener_Taxitarif_1997_2.pdf. In addition to the local authority provides the following additional information about the taxi tariff system, in particular where possible additional surcharges may be added to the fare:

- 1 surcharge if the taxi was ordered via a "taxi-rank-phone" (Standplatztelefon);
- 2 surcharges if the taxi was ordered via call centre (Taxifunkzentrale);
- € 2.00 surcharge for the carriage of 4 or more passengers;
- € 13.00 surcharge if the destination is the airport and the taxi was not pre-ordered;
- Further surcharges e.g. for loading the luggage or for going to the station are prohibited.
- The driver has to inform the passenger about any surcharges added.

Charging anything different than mentioned is an administrative offence.

		Daytime	Nighttime/Weekend
Fixed Fee		€ 3.80	€ 4.30
Variable Fee	0-4,000m	€ 0.20 per 140.7m	€ 0.20 per 123.2m
(Mileage Charge,	4,000-9,000m	€ 0.20 per 184.6m	€ 0.20 per 156.8m
Wait Time Charge)	>9,000m	€ 0.20 per 190.6m	€ 0.20 per 169.5m
	Waiting Time	€ 0.20 per 25.9sec.	€ 0.20 per 25.9 sec.
One Surcharge		€ 1.40	€ 1.40

Table A4: Official Viennese taxi fares

A3 Drivers' nationalities

Ethnicity	*Absolute Frequency*	*Frequency in %*
African	110	19.47
Asian	246	43.54
European	206	36.46
thereby Austrian	***106***	***18.76***
Other	3	0.54
Total	565	100.00

Notes: Note that we clustered certain countries/indications of driver's ethnicity to these categories (e.g. "African" and "Arabian" are clustered to the ethnicity "African"). However, where ever possible (i.e. as controls), we use the full information about the estimated driver's origin.

Table A5: Drivers' nationality

Table A5 summarizes the nationalities of the drivers in our experiment.

A4 Analysis using additional baselines

In this section we present the results relating to the OSM and Uber baseline, excluded from Section 5.

A4.1 OSM Baselines

This section presents results for Section 5, using OSM as a baseline.

Dependent Variable:	*Normalised Distance, OSM*				
Model:	(1)	(2)	(3)	(4)	(5)
Honesty Treatment	-0.039	-0.036	-0.053	-0.038	-0.046
	(0.046)	(0.046)	(0.043)	(0.047)	(0.046)
Dishonesty Treatment	-00	-0.001	-0.018	0.003	-0.015
	(0.044)	(0.044)	(0.044)	(0.045)	(0.043)
Uber Treatment	-0.011	-0.007	-0.028	-0.019	-0.023
	(0.047)	(0.047)	(0.046)	(0.048)	(0.046)
Constant	0.093***	0.091***	0.055***	0.048***	0.041***
	(0.027)	(0.026)	(0.016)	(0.013)	(0.01)
Observations:	486	486	453	453	453
Controls					
Set 1	✓	✓	✓	✓	✓
Set 2		✓	✓	✓	✓
Set 3			✓	✓	✓
Set 4				✓	✓
Set 5					✓

Notes: Robust standard errors in parentheses, clustered by quadruple. Models (1)–(5) are Tobit regressions censored at 1. The presented explanatory variables are dummy variables that take a value of 1 if the observation is taken from that treatment (and 0 otherwise).
***, ** and * denote significance at the 1%, 5% and 10% level.

Table A6: Treatment Effects on Distances - OSM baseline

A4.2 Uber Baselines

This section presents results for Section 5, using the Uber price as a baseline.

A4.3 Google Baselines

To examine this, and determine the level of overcharging relative to our perfectly informed baselines, we compare the observe fare obtained using prime p in quadruple i, $f_{p,i}$ to the fare associated with the journey planned by each of our informed baselines, k, in quadruple i, $f_{k,i}$, where $k \in \{Google\ Maps, OSM, Uber\}$. The overcharging difference is defined as

$$O_{p,i,k} = f_{p,i} - f_{k,i} \tag{1}$$

Table A9 presents the overcharging differences for each treatment, using each of the three baselines. As can be seen, there is overcharging in all treatments, and this is significantly different to zero ($p < 0.01$). Regardless of baseline, similar patterns emerge in the data, although there are no significant differences between treatments ($p > 0.1$ in all cases). Importantly, this implies that our treatments, when compared to a perfectly informed passenger who is never overcharged, *all produce significant levels of overcharging.*

We examine treatment differences further using Tobit regressions, for brevity presenting only those using the Google Maps baseline, and the same sets of controls as those previously defined. As can be seen in Table A10, the coefficients on the treatment dummies are

Dependent Variable:	*Overcharging Difference*				
Model:	(1)	(2)	(3)	(4)	(5)
Honesty Treatment	0.59*	0.398	0.398	0.492	0.435
	(0.342)	(0.284)	(0.284)	(0.313)	(0.313)
Dishonesty Treatment	-0.469	-0.096	-0.096	-0.239	-0.275
	(0.375)	(0.332)	(0.332)	(0.335)	(0.316)
Uber Treatment	0.37	0.345	0.345	0.241	0.598*
	(0.388)	(0.376)	(0.376)	(0.354)	(0.338)
Constant	15.242***	0.088	7.348***	6.612***	4.525***
	(2.308)	(0.108)	(1.497)	(1.262)	(0.82)
Observations:	486	486	453	453	453
Controls					
Set 1	✓	✓	✓	✓	✓
Set 2		✓	✓	✓	✓
Set 3			✓	✓	✓
Set 4				✓	✓
Set 5					✓

Notes: Baseline treatment is taken as the baseline. Robust standard errors in parentheses, clustered by quadruple. Models (1)–(5) are Tobit regressions censored at 0. The presented explanatory variables are dummy variables that take a value of 1 if the observation is taken from that treatment (and 0 otherwise).
***, ** and * denote significance at the 1%, 5% and 10% level.

Table A7: Treatment Effects on Overcharging - OSM Baseline

all not significant at the 5% level. However, as the constant is positive and significant this supports our assertions that there is a significant level of overcharging relative to the informed passenger baseline, but no differences between treatments.

The estimates from Table A10 suggest that, although there is a significant amount of overcharging relative to a perfectly informed passenger in all treatments, there are no treatment differences when comparing the effects of the primes to the fully informed baselines. This observation suggests that the effect of asymmetry in knowledge on overcharging is greater than the effects of the primes; the vast majority of the overcharging we observe relative to the case where there is no overcharging is a result of our testers being ‘foreign’, and being viewed as having limited knowledge.

Dependent Variable:	*Overcharging Difference*				
Model:	(1)	(2)	(3)	(4)	(5)
Honesty Treatment	0.708**	0.419	0.419	0.535	0.459
	(0.338)	(0.301)	(0.301)	(0.333)	(0.338)
Dishonesty Treatment	0.219	0.074	0.074	-0.029	-0.141
	(0.366)	(0.347)	(0.347)	(0.339)	(0.328)
Uber Treatment	0.747*	0.445	0.445	0.357	0.669*
	(0.45)	(0.413)	(0.413)	(0.385)	(0.372)
Constant	12.111***	-0.916***	8.012***	7.162***	4.951***
	(2.113)	(0.139)	(1.725)	(1.399)	(0.887)
Observations:	486	486	453	453	453
Controls					
Set 1	✓	✓	✓	✓	✓
Set 2		✓	✓	✓	✓
Set 3			✓	✓	✓
Set 4				✓	✓
Set 5					✓

Notes: Baseline treatment is taken as the baseline. Robust standard errors in parentheses, clustered by quadruple. The number of observations falls as the number of controls are increased due to missing entries. Models (1)–(5) are Tobit regressions censored at 0. The presented explanatory variables are dummy variables that take a value of 1 if the observation is taken from that treatment (and 0 otherwise).
***, ** and * denote significance at the 1%, 5% and 10% level.

Table A8: Treatment Effects on Overcharging - Uber Baseline

	Baseline	*Honesty*	*Dishonesty*	*Uber*
Overcharging Difference, Google Maps Price	4.13	4.42	4.18	4.46
	(4.43)	(4.14)	(4.1)	(4.62)
Overcharging Difference, Open Street Maps Price	4.42	4.74	4.45	4.75
	(5.03)	(4.71)	(4.71)	(5.11)
Overcharging Difference, Uber Price	3.94	4.27	4.04	4.29
	(5.65)	(5.19)	(5)	(5.5)
Observations:	127	133	137	140

Notes: Normalised distances are calculated by dividing the distance from each treatment by the distance from Google Maps (OSM, respectively). Standard deviations in parentheses.

Table A9: Overcharging Difference - Informed passenger baselines

Dependent Variable:	*Overcharging Difference*				
Model:	(1)	(2)	(3)	(4)	(5)
Honesty Treatment	0.45	0.349	0.349	0.44	0.392
	(0.321)	(0.285)	(0.285)	(0.309)	(0.312)
Dishonesty Treatment	-0.452	-0.121	-0.121	-0.272	-0.288
	(0.349)	(0.319)	(0.319)	(0.323)	(0.306)
Uber Treatment	0.331	0.362	0.362	0.283	0.58*
	(0.375)	(0.372)	(0.372)	(0.346)	(0.335)
Constant	12.002***	0.106	7.06***	6.356***	4.542***
	(1.755)	(0.098)	(1.463)	(1.217)	(0.807)
Observations:	486	486	453	453	453
Controls					
Set 1	✓	✓	✓	✓	✓
Set 2		✓	✓	✓	✓
Set 3			✓	✓	✓
Set 4				✓	✓
Set 5					✓

Notes: Baseline treatment is taken as the baseline. Robust standard errors in parentheses, clustered by quadruple. Models (1)-(5) are Tobit regressions censored at 0. The presented explanatory variables are dummy variables that take a value of 1 if the observation is taken from that treatment (and 0 otherwise).

***, ** and * denote significance at the 1%, 5% and 10% level.

Table A10: Treatment Effects on Overcharging - Google Baseline
